# Severe mental illness and mortality of hospitalized ACS patients in the VHA

**DOI:** 10.1186/1472-6963-7-146

**Published:** 2007-09-18

**Authors:** Mary E Plomondon, P Michael Ho, Li Wang, Gwendolyn T Greiner, James H Shore, Joseph T Sakai, Stephan D Fihn, John S Rumsfeld

**Affiliations:** 1 Cardiology Section, Denver VA Medical Center, Denver CO, USA; 2 Department of Medicine, University of Colorado Health Sciences Center, Denver CO, USA; 3 Health Services Research and Development, Department of Veterans Affairs Puget Sound Health Care Center, Seattle WA, USA; 4 Department of Psychiatry, University of Colorado Health Sciences Center, Denver CO, USA

## Abstract

**Background:**

Severe mental illness (SMI) has been associated with more medical co-morbidity and less cardiovascular procedure use for older patients with myocardial infarction. However, it is unknown whether SMI is associated with increased long term mortality risk among patients presenting with acute coronary syndromes (ACS). We tested the hypothesis that SMI is associated with higher one-year mortality following ACS hospitalization.

**Methods:**

All ACS patients (n = 14,194) presenting to Veterans Health Administration (VHA) hospitals between October 2003 and September 2005 were included. Survival analysis evaluated the association between SMI and one-year all-cause mortality, adjusting for demographics, co-morbidities, in-hospital treatment, and discharge medications.

**Results:**

Overall, 18.4 % of ACS patients had SMI. Patients with SMI were more likely female, younger, Caucasian race, have a history of alcohol abuse, liver disease, dementia, hypertension and more likely to be a current smoker; however, prior cardiac history was similar between the 2 groups. There were no significant differences in cardiac procedure use, including coronary angiogram (38.7% vs. 40.3%, p = 0.14) or coronary revascularization (31.0% vs. 32.3%, p = 0.19), and discharge medications between those with and without SMI. One-year mortality was lower for patients with SMI (15.8% vs. 19.1%, p < 0.001). However, in multivariable analysis, there were no significant differences in mortality (HR 0.91; 95% CI 0.81–1.02) between patients with and without SMI.

**Conclusion:**

Among ACS patients in the VHA, SMI is prevalent, affecting almost 1 in 5 patients. However, patients with SMI were as likely to undergo coronary revascularization and be prescribed evidence-based medications at hospital discharge, and were not at elevated risk of adverse 1-year outcomes compared to patients without SMI.

## Background

Acute Coronary Syndrome (ACS), including unstable angina and acute myocardial infarction (AMI), accounts for over 800,000 hospitalizations yearly [1]. Prior studies have shown that disparities exist for AMI care according to socio-economic status, age, gender, and race. Furthermore, these disparities in AMI care have been associated with differential patient outcomes. For example, women with AMI are less likely to receive acute reperfusion therapy and have higher in-hospital mortality rates compared to men [2-7].

More recently, concerns have been raised regarding potential disparities in AMI care for patients with severe mental illness (SMI), including schizophrenia, mood disorders, anxiety disorders and personality disorders. Prior studies have found that patients with SMI were less likely to receive coronary revascularization and have higher risk of death following AMI [8-10]. The cited reasons for this inequality include increased medical comorbidity, reduced access to medical technology, social isolation, low income, interference with informed consent because of the cognitive symptoms and provider hesitation to aggressively treat SMI patients [8-10]. However, it is unknown whether this disparity exists in a fully integrated system, such as the VHA (Veterans Health Administration), where there is more equal access to medical technology.

Accordingly, the objective of this study was to evaluate the association between SMI and outcomes (1-year all-cause mortality and all-cause mortality or re-hospitalization for AMI) in a national Veterans Health Administration (VHA) cohort of ACS patients hospitalized from 2003 to 2005. We hypothesized that ACS patients with severe mental illness would undergo less coronary revascularization during the index hospitalization and have higher 1-year all-cause mortality and AMI re-hospitalization. The results of this study may have important implications regarding disparities in AMI care and outcomes.

## Methods

Information for this study was collected as part of the VHA External Peer Review Program (EPRP) for quality monitoring and improvement for a variety of medical conditions and procedures, including AMI and unstable angina (UA). Patients with International Classification of Diseases 9^th ^Revision diagnosis codes 410.xx and 411.xx were identified from the VA Patient Treatment File. Working with the EPRP abstraction contractor, West Virginia Medical Institute, the VHA Office of Quality and Performance generated a list of patients that was transmitted to VHA facilities where both paper and electronic medical records were manually abstracted by trained abstractors using standard reporting forms. Abstracted data were then entered into a database maintained by the contractor. Additional details of the study methods have been published [11].

### Subjects

All patients admitted with AMI or UA as documented by standard electrocardiographic criteria, elevated troponin levels, and/or other clinical evidence and discharged from VHA medical centers between 10/1/2003 and 9/30/2005 were included. Patients who were transferred from or to a non-VHA hospital, patients who left against medical advice and patients who died during the index hospitalization were excluded. The analytical cohort for the current study was 14,194 patients.

The primary independent predictor variable of interest was the presence of severe mental illness (SMI). Consistent with other literature, patients were categorized as having SMI if they had any one or more of the following ICD-9 diagnosis codes: Schizophrenia (295.30, 295.10, 295.20, 295.90); Mood disorders (296.3X, 296.40, 296.4X, 296.6X, 296.5X, 296.7, 296.89) However, mood disorders specified as mild or either partial or full remission were not included; Anxiety disorders (300.01, 300.21,300.22, 300.23, 300.3, 309.81, 300.02); Personality disorders (301.0, 301.20, 301.22, 301.7, 301.83, 301.50, 301.81, 301.82, 301.6, 301.4, 301.9) [10].

### Outcome variables

The primary outcome for this study was 1-year all-cause mortality. The secondary outcome was the combined endpoint of all-cause mortality or re-hospitalization for AMI, within 1-year following the index ACS admission. All-cause mortality was defined using the Vital Statistics database, which combines four sources of VA mortality data; VA Beneficiary Identification and Record Locator, in-patient file, Medicare and Social Security Administration. When compared against the National Death Index, the sensitivity was over 98% [12]. Re-hospitalization for AMI was defined using a primary discharge ICD-9 diagnosis code of 410.XX.

### Statistical analyses

Baseline patient characteristics, co-morbidities, presentation factors, hospital treatment and discharge medications among eligible patients were compared between patients with and without SMI using the Chi-square test for categorical variables and t-test for continuous measures. The unadjusted survival was compared between patients with and without SMI using the Kaplan-Meier method. Survival was measured beginning at hospital discharge and censored at the time of death or re-hospitalization for AMI. Differences in event rates were evaluated with the log-rank test. Next, a series of multivariable Cox regression models were constructed to assess the association between SMI and mortality with incremental adjustment for demographic, cardiac, and non-cardiac variables, presentation variables, in-hospital procedures and discharge medications as listed in the Table 1. The same multivariable models were constructed to assess the association between SMI and the combined outcome of all-cause mortality or re-hospitalization for AMI. Because the association between SMI and the outcomes showed no differences in significance or magnitude of effect across the incremental models, only the results of the final model are reported.

**Table 1:** Characteristics of the study population

**Variables**	**No SMI N = 11571(81.6%)**	**SMI N = 2623 (18.4%)**	**p-value**
**Demographic**			
Mean age (SD), yrs	69.6 (11.5)	64.0 (11.7)	< 0.001
Male	11357 (98.1%)	2548 (97.1%)	0.001
Caucasian race	5943 (51.4%)	1553 (59.2%)	< 0.001
BMI (SD)	27.9 (5.9)	28.9(6.4)	< 0.001
**Cardiac**			
Prior AMI	2757 (23.8%)	628 (23.9%)	0.900
Prior CHF	3259 (28.2%)	7317 (27.9%)	0.760
Prior CABG	2509 (21.7%)	502 (19.1%)	0.004
Prior PCI(6 m)	487 (4.2%)	124 (4.7%)	0.237
**Non-Cardiac**			
Alcohol abuse	1037 (9.0%)	725 (27.6%)	< 0.001
Cancer	1098 (9.5%)	174 (6.6%)	< 0.001
COPD	2149 (18.6%)	532 (20.3%)	0.043
Dementia	1016 (8.8%)	967 (36.9.0%)	< 0.001
Lipid disorder	7327 (63.3%)	1655 (63.1%)	0.828
Liver disease	469 (4.1%)	227 (8.6%)	< 0.001
Cerebral Vascular Disease	1072 (9.3%)	242 (9.2%)	0.951
Musculoskeletal	761 (6.6%)	188 (7.2%)	0.274
Renal disease	2547 (22.0%)	508 (19.4%)	0.003
Diabetes	2396 (20.7%)	556 (21.2%)	0.577
Current smokers	3301 (28.5%)	1161 (44.3%)	< 0.001
Hypertension	10155 (87.8%)	2355 (89.8%)	0.004
Peripheral Vascular Disease	3542 (30.6%)	711 (27.1%)	< 0.001
**Presentation**			
Heart rate	85.6 (23.0)	84.9 (22.0)	0.130
Systolic blood pressure	140 (30.0)	138 (29)	0.001
Diastolic blood pressure	76 (18.0)	77 (19)	0.012

BMI, body mass index; AMI, myocardial infarction; CHF, chronic heart failure; COPD, Chronic obstructive pulmonary disease; CABG, coronary artery bypass graft; PCI, percutaneous coronary intervention; COPD, Chronic obstructive pulmonary disease; Cath, Cardiac Catheterization;

In secondary analysis, we assessed the association between SMI and outcomes among the following patient subgroups: unstable angina or acute myocardial infarction, age ≥ 65 or < 65, prior known coronary artery disease, and the subgoups of SMI diagnoses including, schizophrenia, mood or anxiety disorder and personality disorder.

All analyses were done using Stata version 9.0 (StataCorp, College Station, TX). This study was approved by the University of Washington Institutional Review Board and waiver of informed consent was granted.

## Results

Characteristics of the study population are outlined in Table 1. Overall, 18.4% (n = 2,623) of the study population had a diagnosis of SMI. Of the patients with SMI, 65.5% (n = 1718) had a diagnosis of anxiety disorder, 47.1% (n = 1235) had a diagnosis of mood disorder, 15.5% (n = 406) had a diagnosis of schizophrenia, and 11.7% (n = 307) had a diagnosis of personality disorder (not mutually exclusive categories). Patients with SMI were younger, more often Caucasian, and more often female. They were more likely to have a diagnosis of alcohol abuse, liver disease, and dementia, and to be current smokers. Prior cardiac history was similar between the 2 groups, except that patients with SMI were less likely to have had prior CABG surgery.

**Figure 1:**
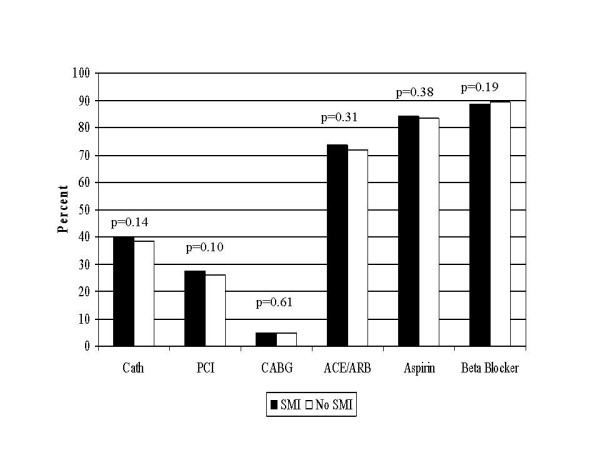
**Comparison of receipt of care SMI and non-SMI patients**. Cath = Cardiac Catheterization ; PCI = percutaneous coronary intervention; CABG = coronary artery bypass graft; ACEI/ARB = angiotensin-converting enzyme inhibitors/Angiotensin II Receptor Blockers;

**Figure 2:**
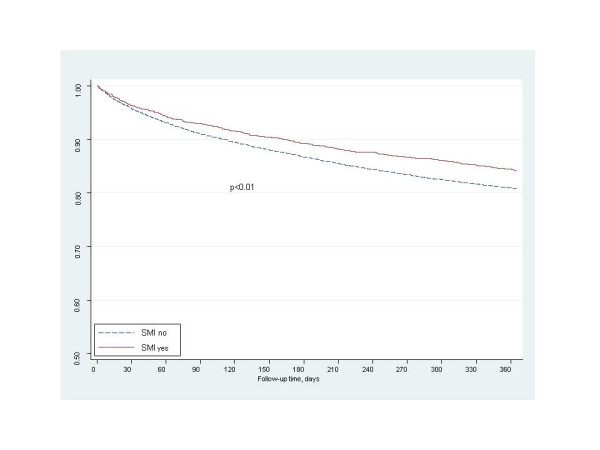
Freedom from 1-year mortality.

**Figure 3:**
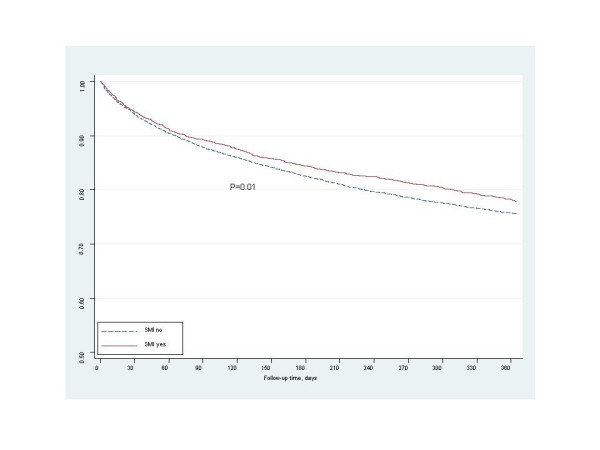
Freedom from 1-year mortality/re-hospitalization.

Among eligible patients, there were no significant differences in the rates of receipt of diagnostic coronary angiogram and coronary revascularization between patients with and without SMI (Figure 1). At hospital discharge, there were similar prescription rates for aspirin, ACE-inhibitor/ARB, and β-blocker medications between the 2 patient groups (Figure 1). In unadjusted analysis, the freedom from event rate was significantly higher among SMI patients compared to those without SMI for 1-year all-cause mortality (84.2% vs 80.9%, p < 0.01) and the combined endpoint of all-cause mortality and re-hospitalization (78.0% vs 75.6%, p = 0.01) for AMI (Figures 2 and 3).

However, after adjustment for demographic, cardiac and non-cardiac co-morbidities, presentation factors, in-hospital procedures and discharge medications, there was no significant association between SMI and all-cause mortality (HR 0.91; 95% CI 0.81–1.02) or the combined endpoint of all-cause mortality and re-hospitalization for AMI, (HR 0.99; 95% 0.90–1.10). The findings were consistent when analyzed by subgroups of SMI ; schizophrenia (0.83; 95% CI 0.60–1.15), mood/anxiety disorder (0.91; 95% CI 0.79–1.06), and personality disorder (1.0; 95% CI 0.68–1.5). Additionally, the association between SMI and outcomes remained consistent when analyzed by different patient subgroups, including unstable angina or myocardial infarction, age ≥ 65 or < 65, and prior known coronary artery disease.

## Discussion

The goal of this study was to assess the association between SMI and 1-year all-cause mortality and AMI re-hospitalization among a national cohort of veterans with ACS. Overall, 18.4% of the study population had a diagnosis of SMI. There were no significant differences in the rates of coronary angiography, PCI or CABG during the index hospitalization or prescription rates of evidence-based discharge medications between patients with and without SMI. Finally, there were no significant differences in risk-adjusted 1-year all-cause mortality or AMI re-hospitalization between ACS patients with and without SMI.

The existing literature on the association between SMI and outcomes following acute myocardial infarction has been mixed. In prior studies, the prevalence of SMI among patients presenting with AMI ranged from 4.7% to 40% depending on the population studied and the definitions used for SMI. While other studies have had a broader definition of SMI and included diagnoses such as other psychoses, alcohol/substance abuse disorders, and/or adjustment reaction as part of their definition, we had a more restricted definition and only included patients with schizophrenia, mood disorders, anxiety disorders and personality disorders [8-10,13,14]. In addition, previous studies have shown that patients with severe mental illness were less likely to undergo diagnostic coronary angiography, and revascularization procedures and had a trend towards higher 1 year mortality following AMI hospitalization [8-10,14]. In contrast, we assessed a contemporary cohort of ACS patients with SMI in a large integrated health care system, used chart abstracted data for risk-adjustment, and performed sensitivity analyses among strata of SMI and patient subgroups. We found that there were no significant differences in the rates of coronary angiography and coronary revascularization between eligible patients with and without SMI and 1-year all-cause mortality and AMI re-hospitalization. These findings suggest that the presence of SMI among patients presenting with ACS does not negatively impact in-hospital care or long-term outcomes in the VHA.

There are several potential explanations for our findings of no difference in outcomes between patients with or without SMI presenting with ACS in the VHA. First, access to care may be less of an issue for SMI patients in the VHA compared to other healthcare settings. Other studies of patients in the VHA have shown no disparities of care and outcomes by race and gender [15,16]. Second, a prior study found that VA physicians were more likely to initiate mental health referral for patients with depression compared to non-VA physicians [17]. One potential explanation is that mental health disorders are common among veterans and because it is a common condition, there may be less of a physician bias in the VHA compared to non-VHA settings. Finally, prior studies have shown that a collaborative care model between primary care and mental health providers for VHA patients with depression is associated with more rapid improvement in symptoms as well as mental health status [18,19]. Additional studies are needed to determine if such a care model for patients with concomitant cardiovascular and severe mental illness will lead to improved patient outcomes.

There are several limitations of this study which should be acknowledged. The results of this study may not be generalizable to all patients with SMI who are hospitalized for ACS because our study cohort comprised of veterans who were predominantly male, older, and had significant comorbidities. Additionally, we were unable to adjust for the veterans socioeconomic status. However, VHA eligibility is determined by disability related to military service or by economic disadvantage [20]. This study included a national cohort of all patients with ACS in the VHA from 2003 through 2005 and therefore represents practice patterns for patients with SMI and ACS in a large integrated healthcare system. Second, we defined SMI using ICD-9 codes and, therefore, could not assess the severity of a patients' mental illness and future studies should assess whether patient outcomes following ACS hospitalization vary according to the severity of the underlying mental disorder. Third, we did not observe a difference in 1-year outcomes following ACS hospitalization between patients with and without SMI, raising the possibility of Type II error. However, we had 99% power to detect an absolute mortality difference of 3.5% between the 2 comparison groups.

## Conclusion

In summary, we found that among ACS patients in the VHA, SMI is prevalent affecting almost 1 in 5 ACS patients. However, patients with SMI were just as likely to undergo coronary revascularization and to be prescribed evidence-based medications at hospital discharge compared to patients without SMI. One-year mortality and re-hospitalization for MI were similar between the two groups. Thus, there is no evidence for disparity in care and outcomes based on the presence of SMI among ACS patients in the VHA.

## Abbreviations

SMI, severe mental illness; ACS, acute coronary syndrome; VHA, Veterans Health Administration; AMI, acute myocardial infarction; EPRP, external peer review program; US, unstable angina; BMI, body mass index; CHF, chronic heart failure; COPD, Chronic obstructive pulmonary disease; CABG, coronary artery bypass graft; PCI, percutaneous coronary intervention; COPD, Chronic obstructive pulmonary disease; Cath, Cardiac Catheterization;

## Competing interests

The author(s) declare that they have no competing interests.

## Authors' contributions

All authors made substantial contributions to conception and design, or acquisition of data, or analysis and interpretation of data. All authors have been involved in drafting of manuscript or revising it critically for important intellectual content; and all authors have given final approval of the version to be published.

## Pre-publication history

The pre-publication history for this paper can be accessed here:


